# Developing cognitive workload and performance evaluation models using functional brain network analysis

**DOI:** 10.1038/s41514-023-00119-z

**Published:** 2023-10-06

**Authors:** Saeed Shadpour, Ambreen Shafqat, Serkan Toy, Zhe Jing, Kristopher Attwood, Zahra Moussavi, Somayeh B. Shafiei

**Affiliations:** 1 Department of Animal Biosciences, University of Guelph, Guelph, Ontario, N1G 2W1 Canada; 2 Intelligent Cancer Care Laboratory, Department of Urology, Roswell Park Comprehensive Cancer Center, Buffalo, NY 14263 USA; 3 Department of Basic Science Education, Virginia Tech Carilion School of Medicine, Roanoke, VA 24016 USA; 4 Department of Biostatistics and Bioinformatics, Roswell Park Comprehensive Cancer Center, Buffalo, NY 14263 USA; 5 Department of Electrical and Computer Engineering & Biomedical Engineering Program and Department of Psychiatry, University of Manitoba, Winnipeg, Manitoba R3T 5V6 Canada

**Keywords:** Quality of life, Learning and memory

## Abstract

Cognition, defined as the ability to learn, remember, sustain attention, make decisions, and solve problems, is essential in daily activities and in learning new skills. The purpose of this study was to develop cognitive workload and performance evaluation models using features that were extracted from Electroencephalogram (EEG) data through functional brain network and spectral analyses. The EEG data were recorded from 124 brain areas of 26 healthy participants conducting two cognitive tasks on a robot simulator. The functional brain network and Power Spectral Density features were extracted from EEG data using coherence and spectral analyses, respectively. Participants reported their perceived cognitive workload using the SURG-TLX questionnaire after each exercise, and the simulator generated actual performance scores. The extracted features, actual performance scores, and subjectively assessed cognitive workload values were used to develop linear models for evaluating performance and cognitive workload. Furthermore, the Pearson correlation was used to find the correlation between participants’ age, performance, and cognitive workload. The findings demonstrated that combined EEG features retrieved from spectral analysis and functional brain networks can be used to evaluate cognitive workload and performance. The cognitive workload in conducting only Matchboard level 3, which is more challenging than Matchboard level 2, was correlated with age (0.54, *p*-value = 0.01). This finding may suggest playing more challenging computer games are more helpful in identifying changes in cognitive workload caused by aging. The findings could open the door for a new era of objective evaluation and monitoring of cognitive workload and performance.

## Introduction

Cognitive workload, which has been identified as a factor impacting learning and performance as well as an impacting on daily activities, is described as mental effort devoted to a task^1^. Increased task demands increase the requirements for already limited working memory (WM) resources, and result in an increased cognitive workload^1^. Long-term memory (LTM) is a relatively unlimited data storage that stores previously acquired information. The efficient interaction between LTM and WM resources enables healthy people to balance the cognitive demands of complicated tasks^1^. When executing the same task, healthy older people continuously showed higher cognitive workloads than younger adults^2^. This observation suggests that an excessive cognitive workload may be caused by the inefficient use of brain resources and may potentially be a sign of cognitive impairment^2^.

Clinical and non-clinical uses for cognitive workload evaluation include improving learning and skill development, enhancing performance in physically demanding professions like aviation^3,4^ and surgery^5^, determining whether commercial video games can delay cognitive decline, and creating models for the early detection of cognitive decline in the elderly^6,7^.

Contradictory findings have been found in studies examining how cognitive activities influence the improvement of executive functions in the brains of healthy aging people. Several studies found a link between engaging in mental exercises, such as playing video games, and physical and cognitive health improvements, especially in elderly people^8–13^. This is encouraging since improving seniors’ cognitive capabilities, such as reasoning, making decisions, and memory, may help prevent or mitigate cognitive decline. However, only performing taught activities improved executive functioning in the brain; evidence for this theory has not been shown by non-trained tasks^14–16^.

West et al. explored the connection between playing video games and tissue growth in different areas of the brain connected to memory and the onset of Alzheimer’s Disease (AD)^11^. They assigned three groups of seniors, 55 to 75 years old, to three different activities for about 6 months: group 1 regularly played a three-dimensional (3-D) video game; group 2 received self-directed digital piano lessons, and group 3 did nothing new. The gaming group had significantly increased gray matter in the hippocampus compared to the piano and inactive groups. The authors concluded that playing video games improved seniors’ cognition and reduced their risk factors for AD^11^. Playing 3-D video games has been associated with improved attention and memory, mental flexibility, and multitasking abilities in older people^12^.

In contrast to these findings, a study by Stojanoski et al. investigated the association between cognitive training and improvement in general cognitive function^17^. They conducted a large-scale online study to see whether brain-training techniques were connected to enhanced cognition. They recruited over 1000 people who practiced a variety of brain-training programs for up to 5 years and assessed their cognition using multiple tests that assessed attention, reasoning, working memory, and planning. Even for the most devoted brain trainers, there was no association between any cognitive performance measure. Additionally, no correlation was found between the length of brain training and any measured cognitive ability^17^.

An evidence-based, objective cognitive workload evaluation approach is required to discover whether engaging in a brain exercise, such as playing computer games, can assist seniors in making the most of their cognitive resources to delay cognitive decline. Currently, primary care providers assess cognitive functioning only when a patient or caregiver voluntarily complains of a cognitive problem^18^. However, underdiagnosis is likely to persist because some patients may not be aware of their mental health status^19,20^ or may be unwilling to talk about it because of the shame and concern of social judgment^21^.

Currently, most techniques for evaluating cognitive workload are quite subjective. Furthermore, the shortcomings of computational algorithms and technical constraints have an impact on the validity of existing objective evaluation methodologies, leading to inconsistent results. It has frequently been suggested that electroencephalogram (EEG) signals could be used to evaluate cognitive workload^5,22,23^. The volume conduction phenomenon, which causes signal leakage from one channel to another, is a significant problem with current EEG research and the validity of the findings^24^.

The purpose of this study was to identify specific brain areas, whose function is responsive to cognitive workload and performance, using high temporal and spatial resolution EEG data. In this study, the impact of volume conduction was alleviated by employing a high-density headset to record EEG data and by processing the signals with spatial filtering methods^25^. The results of this study could have therapeutic implications, after being validated in a larger sample, since they could be used to detect cognitive decline by evaluating changes in cognitive workload.

## Results

### Findings based on Approach A

Search information, strength, temporal network flexibility, integration, recruitment, and PSD features were extracted from 116 EEG signals within 21 BAs at four band frequencies. To evaluate cognitive workload and performance, linear random intercept models were developed using all extracted features. The average temporal flexibility in BA 45 at the beta-band frequencies was associated with the performance (Table 1), and the average search information in BA 47 at the theta-band frequencies was associated with the cognitive workload (Table 2) of participants when conducting Matchboard level 2.

**Table 1:** Results of linear random intercept model analysis for performance evaluation at the Matchboard level 2 (number of samples: 87).

Predictor	Estimate	Standard error	*p*-value
Average temporal flexibility in BA 45 at beta-band frequencies	0.16	0.06	0.007

Subject was a significant random effect (*p*-value = 0.002); pseudo R^2^ = 0.72; MAE = 7.36; RMSE = 9.36.

**Table 2:** Results of linear random intercept model analysis for cognitive workload evaluation at Matchboard level 2 (number of samples: 68).

Predictor	Estimate	Standard error	*p*-value
Average search information in BA 47 at theta-band frequencies	−0.39	0.11	0.001

Subject was a significant random effect (*p*-value = 0.001); pseudo R^2^ = 0.95; MAE = 2.34; RMSE = 3.31.

The average temporal flexibility in BA 9 at the theta-band frequencies, the recruitment in BA 47 at the theta-band frequencies, and the average search information in BA 37 at the gamma-band frequencies were associated with the performance in conducting Matchboard level 3 (Table 3).

**Table 3:** Results of linear random intercept model analysis for performance evaluation at Matchboard level 3 (number of samples: 124).

Predictors	Estimate	Standard error	*p*-value
Average temporal flexibility in BA 9 at theta-band frequencies	0.41	0.15	<0.001
Recruitment feature in BA 47 at theta-band frequencies	−0.28	0.10	<0.001
Average search information in BA 37 at gamma-band frequencies	−1.29	0.34	<0.001

Subject was a significant random effect (*p*-value = 0.01); pseudo R^2^ = 0.55; MAE = 9.43; RMSE = 11.61.

Average search information in BA 45 and the average temporal flexibility in BA 44 at the gamma-band frequencies and BA 7 at the beta-band frequencies were associated with the cognitive workload in conducting Matchboard level 3 (Table 4).

**Table 4:** Results of linear random intercept model analysis for cognitive workload evaluation at Matchboard level 3 (number of samples: 96).

Predictors	Estimate	Standard error	*p*-value
Average search information in BA 45 at gamma-band frequencies	0.51	0.16	0.002
Average temporal flexibility in BA 44 at gamma-band frequencies	−0.36	0.11	0.001
Average temporal flexibility in BA 7 at beta-band frequencies	0.29	0.09	0.002

Subject was a significant random effect (*p*-value = 0.001); pseudo R^2^ = 0.88; MAE = 4.23; RMSE = 6.18.

In this study, our initial feature selection method (Approach A) did not include PSD features. However, this finding does not necessarily imply that PSD is unsuitable or impractical for adoption in evaluations of cognitive workload and performance. Indeed, spectral analysis, which utilizes PSD, has been successfully employed for assessing mental workload in several previous studies^26–30^. Models for evaluating performance and cognitive workload, exclusively utilizing PSD features, were developed according to Approach A. The results of these models are presented in Supplementary Table 1. Associations were observed between the average PSD in BA 45 at beta-band and the performance, and between the average PSD in BA 40 at alpha-band frequencies and cognitive workload, at Matchboard level 2. Similarly, the average PSD in BA 20 and BA 45 at beta-band frequencies was associated with performance and cognitive workload at Matchboard level 3, respectively.

Cognitive workload and performance were not significantly correlated in conducting Matchboard level 2 (−0.13, *p*-value = 0.56) and Matchboard level 3 (−0.17, *p*-value = 0.44). Age and performance were not significantly correlated on the Matchboard level 2 (−0.4, *p*-value = 0.06), and similarly age and cognitive workload were not significantly correlated (0.38, *p*-value = 0.08). There was no significant correlation between performance and age (−0.14, *p*-value = 0.5), in conducting Matchboard level 3; however, there was a significant correlation between age and cognitive workload (0.54, *p*-value = 0.01). Age was not selected as a significant predictor in the random intercept models for cognitive workload and performance in Matchboards level 2 and level 3.

### Findings based on Approach B

To further investigate the effects of integrating features extracted from functional brain networks and spectral analysis on performance and cognitive workload evaluation, the GLMM-LASSO method was applied to all extracted features across four brain cortices and frequency bands. The results are presented in Table 5.

**Table 5:** Results of GLMM-LASSO analysis for performance and cognitive workload evaluation at Matchboard levels 2 and 3 based on approach B (96 features extracted from 116 EEG signals).

Predictors	Estimate	*p*-value
Performance, Matchboard level 2		
Average strength in Parietal cortex, at theta-band frequencies	−34.49	0.014
Random Effect; Subjects’ standard deviation: 106.26		
Number of observations: 82; R^2^: 0.67; MAE:5.61; RMSE: 7.23		
Cognitive workload, Matchboard level 2		
Average temporal network flexibility in Occipital cortex at alpha-band frequencies	1.44	0.034
Average temporal network flexibility in Parietal cortex at theta-band frequencies	−2.19	0.004
Average PSD in Frontal cortex at beta-band frequencies	−3.33	<0.001
Random Effect; Subjects’ standard deviation: 284.2		
Number of observations: 62; R^2^: 0.97; MAE: 1.81; RMSE: 2.29		
Performance, Matchboard level 3		
Average search information in Frontal cortex at beta-band frequencies	−17.93	0.031
Average search information in Temporal cortex at beta-band frequencies	22.7	0.022
Average search information in Frontal cortex at gamma-band frequencies	−19.23	0.021
Average search information in Frontal cortex at alpha-band frequencies	29.09	0.035
Average PSD in Occipital cortex at gamma-band frequencies	9.24	0.029
Average PSD in Parietal cortex at gamma-band frequencies	−11.81	0.049
Random Effect; Subjects’ standard deviation: 33.32		
Number of observations: 111; R^2^: 0.54; MAE: 8.57; RMSE: 10.48		
Cognitive workload, Matchboard level 3		
Average temporal network flexibility in Frontal cortex at theta-band frequencies	1.99	0.024
Average strength in Temporal cortex at beta-band frequencies	−10.13	0.001
Average PSD in Frontal cortex at beta-band frequencies	8.44	0.002
Random Effect; Subjects’ standard deviation: 421.94		
Number of observations: 83; R^2^: 0.95; MAE: 3.44; RMSE: 4.18		

### Findings based on Approach C

To investigate the impact of reduced EEG density, EEG features were extracted from 32 channels, distributed according to the international 10–20 system. The GLMM-LASSO method was then applied to all extracted features across the four brain cortices and frequency bands. This analysis was done to evaluate performance and cognitive workload in Matchboard levels 2 and 3. The results are presented in Table 6.

**Table 6:** Results of GLMM-LASSO analysis for performance and cognitive workload evaluation at Matchboard levels 2 and 3 based on approach C (96 features extracted from 32 EEG signals).

Predictors	Estimate	*p*-value
Performance, Matchboard level 2		
Average temporal network flexibility in Occipital cortex at gamma-band frequencies	−4.14	0.021
Average strength in Occipital cortex at beta-band frequencies	19.4	0.027
Average strength in Temporal cortex at gamma-band frequencies	−16.13	0.016
Average PSD in Temporal cortex at theta-band frequencies	4.13	0.048
Random Effect; Subjects’ standard deviation: 83.33		
Number of observations: 84; R^2^: 0.69; MAE: 5.86; RMSE: 7.30		
Cognitive workload, Matchboard level 2		
Average temporal network flexibility in Occipital cortex at beta-band frequencies	2.36	0.008
Average search information in Parietal cortex at beta-band frequencies	6.41	0.003
Average search information in Frontal cortex at gamma-band frequencies	−5.06	0.012
Average search information in Occipital cortex at gamma-band frequencies	3.68	0.045
Average strength in Parietal cortex at alpha-band frequencies	25.73	0.029
Average strength in Occipital cortex at alpha-band frequencies	−13.87	0.021
Average strength in Parietal cortex at gamma-band frequencies	−9.02	0.013
Average strength in Parietal cortex at theta-band frequencies	−39.66	0.001
Average strength in Frontal cortex at theta-band frequencies	14.67	0.006
Average strength in Occipital cortex at theta-band frequencies	23.71	<0.001
Average PSD in Occipital cortex at alpha-band frequencies	−3.99	0.033
Random Effect; Subjects’ standard deviation: 212.56		
Number of observations: 63; R^2^: 0.94; MAE: 1.82; RMSE: 2.41		
Performance, Matchboard level 3		
Average temporal network flexibility in Frontal cortex at beta-band frequencies	5.57	0.033
Average temporal network flexibility in Parietal cortex at gamma-band frequencies	8.31	0.026
Average temporal network flexibility in Occipital cortex at gamma-band frequencies	−6.79	0.002
Average search information in Parietal cortex at theta-band frequencies	8.61	0.025
Average strength in Temporal cortex at gamma-band frequencies	−8.69	0.038
Random Effect; Subjects’ standard deviation: 30.77		
Number of observations: 118; R^2^: 0.57; MAE: 7.52; RMSE: 9.34		
Cognitive workload, Matchboard level 3		
Average temporal network flexibility in Parietal cortex at alpha-band frequencies	2.67	0.024
Average search information in Frontal cortex at beta-band frequencies	4.11	0.001
Average strength in Occipital cortex at alpha-band frequencies	−3.88	0.025
Average PSD in Parietal cortex at alpha-band frequencies	−12.27	0.027
Average PSD in Frontal cortex at alpha-band frequencies	32.17	0.001
Average PSD in Temporal cortex at alpha-band frequencies	−13.13	0.013
Average PSD in Occipital cortex at beta-band frequencies	−5.71	0.014
Average PSD in Frontal cortex at theta-band frequencies	−22.65	0.005
Random Effect; Subjects’ standard deviation: 474.54		
Number of observations: 89; R^2^: 0.96; MAE: 3.05; RMSE: 4.07		

## Discussion

The EEG data recorded from 26 participants conducting two cognitive tasks on a robot simulator were used to extract search information, strength, temporal network flexibility, integration, recruitment, and PSD features in 21 BAs at four band frequencies. Features were used to develop linear models to evaluate cognitive workload and performance.

### Matchboard level 2

Matchboard level 2 requires conscious focus on the characters below Matchboard doors, finding a logical pattern between characters and doors, and memorizing the place of characters below doors.

Average temporal network flexibility in BA 45 at the beta-band frequencies was positively associated with the performance in Matchboard level 2. The BA 45 was proposed, by functional MRI studies, to be involved in working memory^31,32^ and brain oscillations at the beta-band frequencies have shown a connection with conscious thought and logical thinking^33^. The selection of this feature is in line with the nature of Matchboard level 2, which needs WM loading and logical reasoning. This result may indicate that the brain is able to access information from WM resources, to process logical reasoning relevant to cognitive tasks, better if the temporal network flexibility in BA 45 is higher at beta-band frequencies. As a result, performance in conducting cognitive task improves.

Average search information in BA 47 at theta-band frequencies was negatively associated with cognitive workload. The functional MRI studies showed that BA 47 area is involved in WM^31,34^ and executive functions^35^. Brain activity oscillations in the theta-band frequencies have shown a relationship with the processing of new information, creativity, and intuition^36^. This result may suggest that retrieving information from WM resources, to process new information and create executive commands, is less efficient and may result in cognitive overload if search information in BA 47 is higher.

Results showed that performance in conducting straightforward Matchboard can be evaluated by only one feature. Similarly, only one feature was needed and sufficient to evaluate cognitive workload. However, the pseudo R^2^ was lower for performance (0.72) compared to that for cognitive workload (0.95). It may imply that the extracted features can better explain cognitive workload variations than performance.

### Matchboard level 3

Matchboard level 3 is a challenging task where participants should properly manipulate three instruments with minimum collision and force. To complete this task successfully, participants should be aware of the status of each instrument, memorize the locations of characters below Matchboard doors, and make appropriate decisions to choose instrument and character. More features were needed to predict performance and cognitive workload in Matchboard level 3. It might demonstrate how challenging this task is compared to Matchboard level 2.

The average temporal network flexibility in BA 9 at theta-band frequencies was positively associated with the performance. Functional MRI studies showed that BA 9 area is involved in memory encoding and recognition^31,37,38^, memory retrieval^38–40^, working memory^41–43^, executive functions such as executive control of behavior^44^, inferential reasoning^45–47^, decision making^48^, and error processing/detection^49^. The selection of this feature as a performance predictor complies with the requirements for the successful completion of Matchboard level 3. The significant role of flexibility in BA 9 in the performance evaluation model may indicate that (1) conducting Matchboard level 3 requires reasoning, decision making, and retrieving information from WM resources; and (2) the brain retrieves information, to process new stimulations related to cognitive task and make proper decisions, more efficiently if BA 9 is more flexible.

Additionally, performance in the Matchboard level 3 was negatively associated with the recruitment feature in BA 47, which according to functional MRI studies, is involved in working memory^31,34^ and executive functions^35^, at theta-band frequencies. Greater recruitment in BA 47 suggests a better connection within this area than a connection between this area and other BAs. The BA 47 is engaged in WM and executive functions, so it should effectively communicate with other areas to acquire updated inputs and process stimulation information related to executive functions. Hence, greater recruitment of BA 47 reduces task performance.

Performance was negatively associated with search information of BA 37, which is involved in processing visual motion^50–52^ and structural judgments of familiar objects^53^, in the gamma-band frequencies. The gamma-band frequencies have shown a connection with perception and cognitive processes^54^, attention and working memory processes, and information integration^55–60^. This result may suggest that performance decreases if access to BA 37 resources, to process perceptual and cognitive processes, demands a higher amount of information.

Additionally, the higher search information value in BA 45, which contributes to working memory, at gamma-band frequencies was associated with more cognitive workload. This may indicate that more information is required to access WM resources in this area, in order to process perceptual and cognitive functions, resulting in a higher allocation of working memory resources and cognitive overload.

Average temporal network flexibility in BA 44 at gamma-band frequencies was negatively associated with cognitive workload. Functional MRI studies showed that BA 44 is involved in working memory^31,32,61^, goal-intensive processing^62^, and object manipulation^63^. As a result, this finding could suggest that a more flexible BA 44 facilitates information retrieval that is more effective at processing cognitive and perceptual tasks, freeing up WM resources.

Average temporal network flexibility in BA 7 at beta-band frequencies was positively associated with cognitive workload in Matchboard level 3. Functional MRI studies showed that BA 7 is involved in processing tool-use gestures^64,65^, bimanual manipulation^66^, and tactile localization and tactile recognition^67^. The selection of this feature is in line with the characteristics of this task and its required skills. This finding might indicate that the BA 7 should be less flexible (more stable) in processing logical functions and bimanual manipulation for a more efficient allocation of mental resources.

Similar to Matchboard level 2, the pseudo R^2^ was lower for performance (0.55) compared to that for cognitive workload (0.88). It confirms that extracted features are better predictors of cognitive workload rather than performance.

### Relationship between age and cognitive workload

Cognitive workload was correlated with age (0.54, *p*-value = 0.01) in conducting Matchboard level 3. However, there was no correlation between age and cognitive workload in conducting Matchboard level 2. It might be because Matchboard level 2 is a straightforward task and can be completed by adult people of any age. This result may suggest that playing difficult computer games is recommended for detecting changes in cognitive workload caused by aging.

### Combining spectral and network features for cognitive workload and performance evaluation

Both PSD and functional brain network features independently demonstrated robust abilities in assessing performance, particularly in evaluating cognitive workload (Tables 1–4, and Supplementary Table 1). PSD features evaluated performance and cognitive workload in Matchboard level 2 with respective R^2^ values of 0.74 and 0.96, while in Matchboard level 3, these values were 0.46 and 0.85. Functional brain network features could assess performance and cognitive workload in Matchboard level 2 with R^2^ values of 0.72 and 0.95 and in Matchboard level 3 with R^2^ values of 0.55 and 0.88. Notably, functional brain network features showed to be better predictors when evaluating performance and cognitive workload in more complex tasks, as seen in Matchboard level 3.

The integration of PSD and functional brain network features enhanced the performance of cognitive workload evaluation models (Approach B; Table 5). With the inclusion of these features, the models achieved an R^2^ value of 0.97 for the cognitive workload evaluation of Matchboard level 2, and 0.95 for level 3. These results represent improvements over the previous scores of 0.95 and 0.88, respectively, achieved without the involvement of PSD features, following Approach A (Tables 2 and 4, respectively). Similarly, the outcomes present an improvement from the previous scores of 0.96 and 0.85, respectively, attained without employing functional brain network features, also following Approach A (Supplementary Table 1).

PSD helps understand the power contribution of different brain rhythms (like alpha, beta, gamma-band frequencies), which are often linked with different cognitive states. The outcomes from Approach B highlight the substantial value that PSD features add to the evaluation of cognitive workload. For instance, the average PSD within the beta-band frequencies of the frontal cortex was a significant predictive factor for cognitive workload at both Matchboard levels 2 and 3 (Table 5). Furthermore, when evaluating performance and mental workload based solely on PSD features, associations were found between the average PSD in BA 45 at beta-band and the performance, and between the average PSD in BA 40 at alpha-band frequencies and cognitive workload, at Matchboard level 2. Likewise, an association was observed between the average PSD in BA 20 and BA 45 at beta-band frequencies and both performance and cognitive workload at Matchboard level 3, respectively (Supplementary Table 1). Without incorporating PSD features and focusing solely on functional brain network features, such important insights would remain undiscovered.

While the PSD provides useful insights into overall electrical activity in the brain, it might not fully capture the complexities of cognitive workload. On the other hand, functional brain network features capture the dynamics of interaction and communication between different brain regions over time, crucial for performing cognitive tasks^68–73^. Functional network features such as “average temporal flexibility” and “average search information” provide insight into the dynamics of brain network interactions, such as how easily nodes switch between communities (temporal flexibility) or how much information a node gathers from the rest of the network (search information). These network-level dynamics are not captured by PSD features. These features incorporate complex network measures, encapsulating higher-level organizational principles of the brain. In the present study, the important insights derived from the Approach A analyses would be missed if we exclusively utilized PSD, without considering the pivotal role of functional brain network features.

While PSD features provide valuable insights into cognitive workload, relying solely on them may overlook critical information about the collaboration among different brain regions, the efficiency of information flow in the brain network, and the adaptability of the brain network under varying cognitive workload conditions^68–73^. PSD and functional brain network features are complementary to each other. By integrating these two distinct types of features, a more comprehensive understanding of brain functionality can be achieved. Such a combination has the potential to enhance the performance of models used to evaluate cognitive workload, particularly for challenging tasks, therefore offering a more robust and detailed understanding of these processes.

### Impact of reduced EEG electrode density on performance and cognitive workload evaluation

Results showed that a reduced EEG density still provided considerable insight into predicting cognitive workload and performance. Despite this reduced density, the predictors still showed a significant relationship with performance and cognitive workload at Matchboard levels 2 and 3. This is evidenced by the statistical significance of the predictors in the GLMM-LASSO analyses, as well as the relatively high R^2^ values indicating that the predictors could explain a substantial proportion of the variation in the outcomes. This suggests that while higher-density EEG may provide more detailed information, lower-density systems can still yield valuable data. The results obtained from the 32-channel system were still informative, indicating that significant and useful information can be extracted from these lower-density systems.

However, the results from the lower-density EEG (Approach C) were not identical to those from the higher-density EEG (Approach B). Different features and cortices were significant in each case. This suggests that while lower-density EEG can provide useful information, the specific insights it offers may differ from those obtained from a higher-density system. Although high-density EEG may provide more detailed information and facilitate the detection of more complex patterns, lower-density systems can still generate significant and valuable insights.

The models generated from lower-density EEG data incorporated a greater number of features compared to those derived from high-density EEG data. This is displayed by the cognitive workload evaluation model for Matchboard level 2, where the low-density EEG model incorporated 11 features and achieved an R^2^ value of 0.94. In contrast, the corresponding high-density EEG model incorporated only three features, with a slightly higher R^2^ value of 0.97. This comparison suggests that when working with lower-density EEG data, a wider range of features spread across various brain cortices might be necessary to effectively identify patterns associated with performance and cognitive workload. Hence, the nature of the data acquisition method influences the complexity of the models required to capture significant brain activity and its relationship with cognitive performance and workload.

Study strengths: Several studies proposing EEG data to evaluate mental workload use features that are extracted by only spectral analysis of EEG signals rather than considering the dynamic changes in the functional brain network^26–30^. Limited studies showed a relationship between the human brain’s network flexibility and the individual cognitive state^74,75^, learning^75^, mood^74^, cognitive control^76^, and mental workload in robot-assisted surgery^5^ and the relationship between integration and recruitment in learning^77,78^. However, this study investigated the association between cognitive workload, performance, functional brain network features, and PSD features in conducting cognitive tasks. The developed models could be used to evaluate cognitive workload and performance and might be used to identify early warning signals of cognitive decline if they have been validated in a larger sample, especially elderly persons.

Limitations of the study: Although this study’s results are encouraging, there are several limitations. There were only 26 participants, and the majority were young, healthy, and well-educated. Only three participants were above 60 years old. The proposed models should be tested on data from the older population, those with cognitive problems, and people representing different education levels conducting computer games with different complexity levels. Finally, although SURG-TLX is a well-established cognitive workload assessment tool, it relies on the subject’s self-report.

Application of results: We described the role of EEG features in various BAs and band frequencies, which represent the dynamic nature of neural processes during a cognitive task, in performance and cognitive workload level. Our study demonstrates the importance of combining dynamic network neuroscience approaches and spectral analysis in performance and cognitive workload evaluation. Results showed that average search information, average temporal network flexibility, average strength, and PSD were the main features associated with participants’ performance and cognitive workload. Understanding the relationship between extracted features and cognitive workload should improve our understanding of cognitive disorders. The results of this study could have therapeutic implications, after being validated in a larger sample. The results could be used to detect cognitive decline by evaluating changes in cognitive workload in follow-up studies. We will explore the development of follow-up studies with a larger and more diverse sample, including a greater number of elderly participants in the future. This will help validate the results and enhance the generalizability of the findings.

## Methods

This study was conducted in accordance with relevant guidelines and regulations and was approved by the Roswell Park Comprehensive Cancer Center’s Institutional Review Board (IRB; I-241913). The IRB issued a waiver of documentation of consent. Participants were given a research study information sheet and provided verbal consent.

**Fig. 1:**
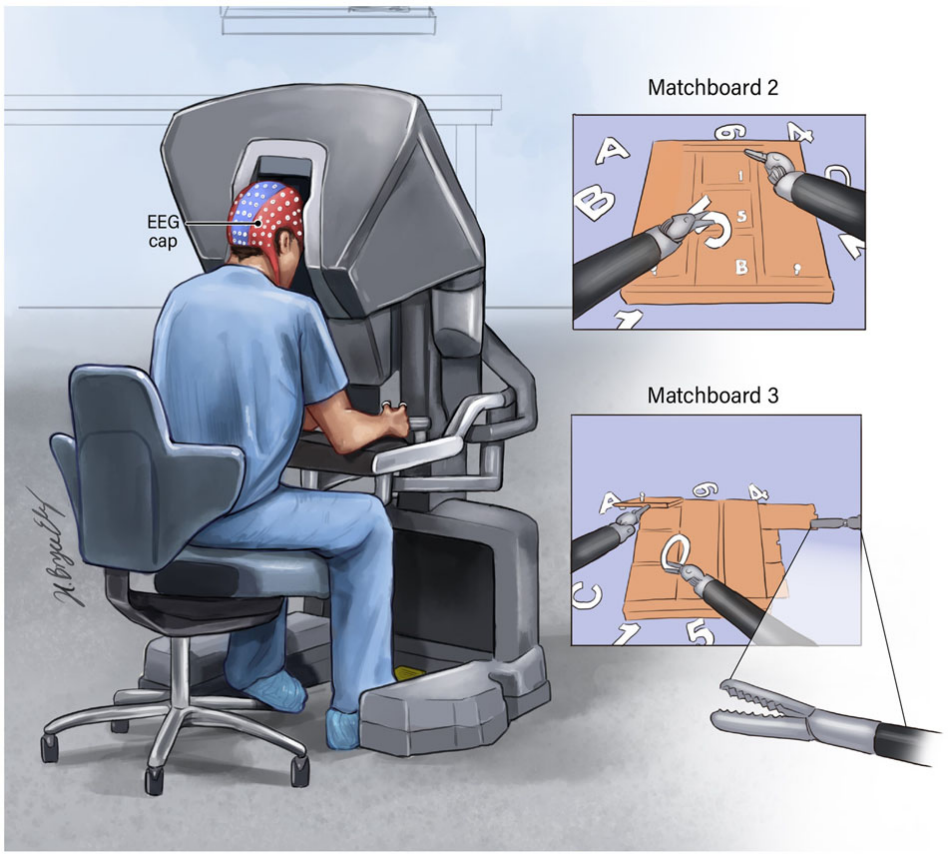
Experimental setup. Illustration of EEG recording from participants who used the da Vinci surgical simulator to conduct two psychomotor cognitive tasks. This figure was developed by the ATLAS illustrator team at RPCCC, using the Adobe Illustrator software.

Twenty-six participants, comprised of 18 males and eight females with an average age of 35.5 years (standard deviation: 11.7 years), conducted two psychomotor cognitive tasks (Matchboard with complexity levels 2 and 3), and EEG data were recorded at a constant rate of 500 Hz using a 124-channel EEG headset (AntNeuro®; Fig. 1), with Cz as the reference channel^79,80^. Participants included premedical students, scientists, surgical residents and fellows, and surgeons. The EEG data were used to extract search information, strength, temporal network flexibility, integration, recruitment, and Power Spectral Density (PSD) features at four band frequencies and 21 Brodmann’s areas (BA). The extracted features were used to evaluate performance and overall cognitive workload using linear models.

The da Vinci® Skills Simulator™ (developed in collaboration with Mimic® Technologies, Inc. Seattle, WA, USA) has two instruments attached to mechanical arms and a camera arm. The participant operates the arms while sitting at a computer console (Fig. 1). The tasks and software developed for this simulator system are similar to well-known computer video games that let players experience virtual reality.

### Tasks and the purpose of conducting each task

There are three levels of complexity for the Matchboard task in the da Vinci simulator program; level 1 is the most straightforward, and level 3 is the most challenging. As all participant performance scores were high (>70) and similar, data for Matchboard level 1 were not analyzed. Data for Matchboards level 2 and level 3 were used in the study. The goal of these tasks, which resembled video games, was to improve psychomotor and cognitive abilities. The tasks involved several cognitive domains, including attention, memory, executive cognitive function, and visuospatial ability^81^. Objects with shapes like numbers and letters appeared around a Matchboard with corresponding character-shaped recesses. In Matchboard level 2, three-panel doors were blocked by the Matchboard. Participants were instructed to retract the panel doors with one instrument while placing the characters in the proper bins with the other. In Matchboard level 3, three sliding doors and three swinging panel doors were blocked by the Matchboard. This task required switching between three instrument arms to move multiple doors. Participants had to retract one of the sliding doors using one of the three instruments before opening the swinging door with the other instrument’s arms to reveal the character bin below. Participants placed the appropriate character in the bin.

### Performance score

Simulator program provided performance scores based on several weighted metrics. The simulator metrics included *time to complete the task*: the total amount of time the user spent on the task (measured in seconds; weight: 17.54); *economy of motion*: total distance traveled by all instruments (measured in centimeters; weight: 17.54); *instrument collisions*: total number of instrument-on-instrument collisions (weight: 17.54); *excessive instrument force*: total time an excessive instrument force was applied above a prescribed threshold force (measured in seconds; weight: 8.77); *instruments out of view*: total distance traveled by instruments outside of the user’s field of view (measured in centimeters; weight: 17.54); *master workspace range*: radius of user’s working volume on master grips (measured in centimeters; weight: 3.51); and *drops*: number of times an object was dropped in an inappropriate region of the scene (weight: 17.54).

The simulator assigned a single score between 0 and 100, where 0 denoted no acceptable performance to complete the simulated task and 100 denoted performance that met all necessary standards.

### Cognitive workload

At the end of each exercise, participants completed the Surgery Task Load Index (SURG-TLX) questionnaire to assess their cognitive workload. The SURG-TLX is a six-domain tool that provides a measure of the perceived cognitive workload^82^. The domains are *Mental demands*: level of mental effort required to complete the task; *Physical demands*: level of physical effort required to complete the task; *Temporal demands*: level of rush in completing the task; *Task complexity*: level of difficulty of the task; *Situational stress*: level of anxious felt during the task completion; and *Distractions*: level of environmental distractions. A scale of 1 to 20 is used for each domain, with 1 designating the minimum and 20 the highest. The scores for the six domains were added to calculate the overall cognitive workload score.

### EEG pre-processing

Due to the poor quality of signals recorded from the F8, POz, AF4, AF8, F6, FC3, M1, and M2, those signals were excluded from this study. EEG artifacts of the remaining 116 channels were corrected by blind source separation and a topographical Principal Component Analysis using the advanced source analysis (ASA) framework. The framework is developed by ANT Neuro Inspiring Technology Inc., Netherlands. EEG artifact correction was done in five steps: (1) EEG data were re-referenced to the “common average reference,” which is the average of all scalp channels involved in the study^83^. (2) A 60 Hz notch filter was applied to remove the line noise. (3) The EEG data were filtered with a band-pass filter (0.2–250 Hz) and a filter steepness of 24 dB/octave. (4) Artifacts related to facial and muscle activity were detected and decontaminated by ASA. Then, individual segments of the EEG data were visually inspected for those artifacts^83^. (5) The spatial Laplacian technique was applied to the decontaminated signals^84^. The Surface Laplacian method emphasizes sources at small spatial scales to alleviate the effect of volume conduction on coherence calculations^25^. After being decontaminated from artifacts, EEG data were used to extract the search information, strength, temporal network flexibility, integration, recruitment, and PSD features at theta (4–8 Hz)^85^, alpha (8–12 Hz)^86^, beta (13–35 Hz)^87^, and gamma (35–65 Hz)^88^ band frequencies, and 21 Brodmann areas (BA).

Each EEG channel was assigned to a specific BA based on their position that was roughly above each BA^89^. The BA that each EEG channel belongs to was identified using the Brodmann’s Interactive Atlas (http://www.fmriconsulting.com/brodmann/Interact.html) and the Brain master software (http://www.brainm.com/software/pubs/dg/BA_10-20_ROI_Talairach/). The 116 EEG channels were assigned to the 21 Brodmann areas (Table 7).

**Table 7:** List of EEG channels roughly above each Brodmann area.

BA	Channels	BA	Channels	BA	Channels
1	C4, CCP4h	2	C3, CP3, CCP3h, CPP3h	5	Cz, CP1, CP2, C1, C2, CCP1h, CCP2h, CPP1h, CPP2h
6	FC1, FC2, FCz, FC4, FCC3h, FCC4h, FCC2h, FCC1h	7	Pz, P1, P2	8	F4, F3, Fz, F1, F2, AFF1, AFF2, FFC3, FFC4, FFC1, FFC2
9	AF3, AFz	10	AFp3h, AFp4h, FPz, FP3, FP4, FP1, FP2	18	O1, O2, I1, I2, OI1h, OI2h, POO9h, POO10h
19	PPO2, PPO1, POO3h, POO4h, PO3, PO4, PO7, PO8	20	FT9, FT10, PO9, P9, FTT9h, FTT10h, PPO9h	21	TP7, TP8, TPP10h, TTP8h, TPP7h, TPP8h, T8, TPP9h
37	PO10, P10, PPO10h, P7, P8	39	P3, P4, P5, P6, PPO5h, PPO6h	40	CP5, CP6, CP4, CPP5h, CPP6h
41	C6, CCP6h	42	CCP5h, TTP7h, T7, C5	44	FC6, FC5, FCC6h
45	FFT8h, FFT7h	46	AFF5h, AFF6h, AF7, F5, FFC5h, FFC6h, FCC5h	47	FTT7h, FTT8h, F7, FT7, FT8

### Extraction of search information and strength features

**Fig. 2:**
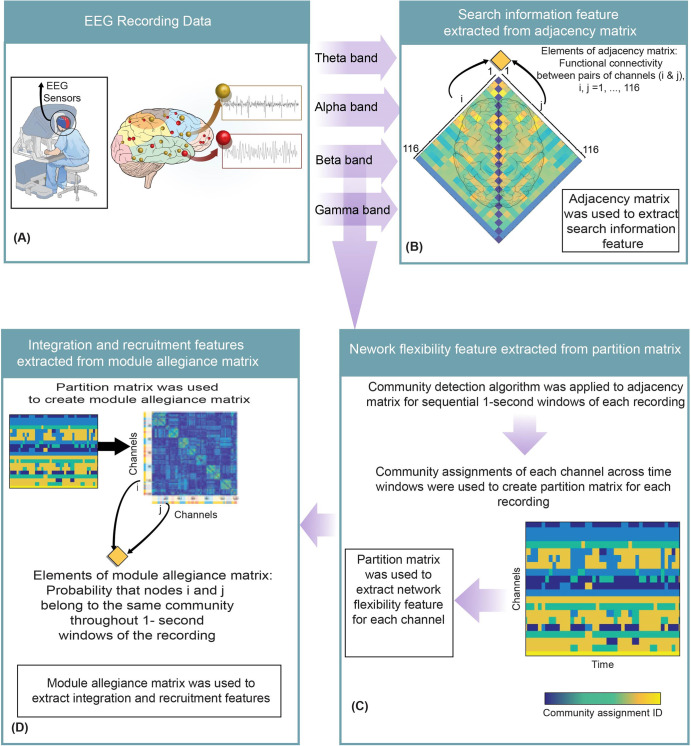
Representation of EEG feature extraction steps. **A** The EEG data were recorded using a high-density EEG headset. **B** Adjacency matrices at different band frequencies were extracted using coherence analysis. Adjacency matrix of each recording was used to extract the search information feature. **C** A community detection algorithm was applied to adjacency matrices associated with 1-s windows throughout a recording. The community assignment for each channel across windows created a partition matrix for each recording and was used to extract temporal network flexibility for each channel. **D** The partition matrix was used to extract a module allegiance matrix for each recording which was then used to extract integration and recruitment features.

Search information is the amount of information (measured in bits) required to pass the shortest, and presumably the most efficient path between two nodes of a network^90–93^. Search information feature was extracted using the adjacency matrix, commonly known as the functional brain network, of each EEG recording^90–92^ and the Brain Connectivity Toolbox (https://sites.google.com/site/bctnet/measures) (Fig. 2A, B). The adjacency matrix is a network that mathematically illustrates the functional connections between various brain areas involved in information processing^94^. Entries of this matrix reflected the weight of connections between various EEG channels i and j ($\Gamma =({{\Gamma}}_{{\rm{ij}}})\in {\Re}^{{\rm{N}}\times {\rm{N}}}$; i and j ranged from 1 to N, where N is the number of EEG channels). Those entries were calculated using the coherence analysis.

Finally, a total of 84 search information features were generated after calculating the average of the extracted search information for channels inside each BA (i.e., 21 BAs and four band frequencies).

The average functional connection weight of channels within each BA was calculated and considered as the strength in that BA. Finally, a total of 84 strength features were generated after calculating the average of the extracted strength for channels inside each BA (i.e., 21 BAs and four band frequencies).

### Extraction of temporal network flexibility feature

The temporal network flexibility (f) of each network node is proportional to the number of times the node changed its network community assignment over time^74^. A network community is described as a subset of network nodes with denser connections between themselves than to other nodes in the network^95^. Temporal network flexibility has been proposed as a functional brain network feature that changes by learning^96^, surprise, and fatigue^97^. This feature has also been proposed for evaluating the mental workload of surgeons conducting surgical tasks^5^.

To calculate the temporal network flexibility feature first adjacency matrix (i.e., functional brain network) was extracted for every one-second window of EEG data recording (Fig. 2C). Then modularity metric associated with each adjacency matrix was extracted using the “community Louvain” function of the Brain Connectivity Toolbox. This metric measures how well nodes are assigned to communities. To detect the network communities, modularity was maximized using a Louvain-like locally “greedy” algorithm^98,99^. This process was repeated 100 times in a consensus iterative algorithm to identify a single consistent representative partition from all partition sets, based on statistical testing in comparison to the ‘Newman–Girvan (NG)’ null network^99,100^. The output of modularity maximization is the community assignment of EEG channels for each 1-s window EEG. The community assignment of each EEG channel is the community that the EEG channel was assigned to (e.g., if five communities were detected for an adjacency matrix, the community assignment of each node is an integer from one to five). The community assignments of EEG channels across 1-s windows were used as elements of the partition matrix $A\in {{\mathfrak{R}}}^{\text{NXT}}$. The elements of the partition matrix ${A}_{i,t}\in \left\{1...g\right\}$ displayed the communities (g) to which brain area i (EEG channels; 1 to N, where N = 116) was assigned at time t (second; t = 1 to T, where T denotes recording duration).

Finally, the partition matrix was used in the flexibility function of Network Community Toolbox (http://commdetect.weebly.com/)^75^ to calculate the temporal network flexibility of each channel as Eq. 1.

1 $${f}_{i}=1-\frac{1}{T-1}\mathop{\sum }\limits_{t=1}^{T-1}\delta ({A}_{i,t},{A}_{i,t+1})$$

The number of times that brain area i changed its community assignment across successive 1-s time windows was measured by the temporal network flexibility of channel i. Low (high) temporal flexibility indicated that the corresponding community assignment of each EEG channel area was stable (changing) across time windows^74,75^.

Finally, the average of the extracted temporal network flexibility for channels within each BA was calculated, resulting in a total of 84 temporal network flexibility features (i.e., 21 BAs and four band frequencies).

### Extraction of integration and recruitment features

Recruitment of a node is described as the “average probability that this node is in the same network community as other nodes from its own system”^77,101^. BAs were considered as systems in this study. Integration of a node corresponds to the “average probability that this node is in the same network community as nodes from other systems”^77,101^. Integration and recruitment have been proposed as functional brain network features that change through practice and learning^77,78^.

Integration and recruitment features were extracted using Module Allegiance Matrix (MAM), which was developed using the partition matrices. Element (i,j) in the MAM represents the probability that nodes ‘i’ and ‘j’ belong to the same community (Fig. 2D) throughout the time of a recording. The interaction strength (I), between two systems C_k1_ and C_k2_, can be defined as the average probability of pairs of channels belonging to the same community, where one electrode belongs to the first system and the second electrode belongs to the second system:

2 $${I}_{k1,k2}=\frac{\sum _{i\in {C}_{k1}j\in {C}_{k2}}{P}_{{ij}}}{\left|{C}_{k1}\right|{\rm{|}}{C}_{k2}{\rm{|}}}$$

where |C_k_ | is the number of nodes in the system C_k_, and k_1_ ≠ k_2_. Note that the average recruitment of a single system to the task can be calculated by letting k_1_ = k_2_ in Eq. (2). The average integration between two separate systems is calculated using the normalized interaction between the two systems (k_1_ ≠ k_2_). Integration and recruitment features were extracted using the MAM matrix and the Network Community Toolbox (http://commdetect.weebly.com/)^77,101^. The P_ij_ in Eq. (2) are elements of MAM.

Finally, the average of the extracted integration and recruitment for channels within each BA was calculated, resulting in a total of 84 integration features (i.e., 21 BAs and four band frequencies) and a total of 84 recruitment features.

### Extraction of power spectral density features

Short Fast Fourier Transform (SFFT) with a one-second Kaiser moving window was used to calculate the power spectral density (PSD) of EEG signals. A 50% overlap was considered for Kaiser moving window. Spectral band power features (henceforth PSD features) were extracted by averaging spectral band power within the standard band frequencies (i.e., theta, alpha, beta, and gamma) for channels within each BA, resulting in a total of 84 PSD features.

### Understanding performance and cognitive workload: the potential insights from functional brain network and PSD features

The functional brain network features enhance our understanding of performance and cognitive workload by providing a range of valuable insights, including:^68–73^ (1) Inter-regional communication: Brain regions communicate with each other to support cognition. Functional connectivity measures can provide insights into the strength and patterns of these interactions, which are crucial for understanding cognitive workload and performance; (2) Efficiency and network topology: Functional brain network analysis can reveal the efficiency of information transfer within the brain. These factors might be relevant for cognitive workload and performance; (3) Network dynamics: Functional brain networks exhibit dynamic changes over time. These changes might be associated with shifts in cognitive state, task demands, or learning processes, and could be missed by analyses that only consider the PSD; (4) Specific cognitive functions: Different cognitive functions are associated with varying patterns of functional connectivity. Analyzing functional brain network features could provide specific insights about the types of cognitive processes that are engaged; (5) Resilience and flexibility: Functional brain network analysis can also provide insights about the brain’s resilience (how well it can handle disruptions) and flexibility (its capacity to reconfigure its connections in response to changing task demands). These factors could be important for understanding individual differences in cognitive workload.

The PSD features provide an understanding of the overall power distribution within specific frequency bands, which is an important factor for understanding cognitive workload and performance.

### Statistical analysis

Extracted features were used as independent variables to develop models for evaluating cognitive workload and performance. The goal was to find the features that are associated with cognitive workload and performance among different participants. Additionally, the correlation between performance, cognitive workload, and participants’ age was determined using the Pearson correlation. All tests were two-sided, and the statistical significance level was 0.05. All statistical analyses were performed with SAS® (version 9.4, SAS Institute Inc., Cary, NC, USA). Three approaches were considered to develop performance and cognitive workload evaluation models.

#### Approach A

Seven-fold cross-validation was used to reduce individual effects in detecting important features (i.e., predictors). Forward feature selection was used to identify the possible predictors. Variables selected at least twice by cross-validation were considered as possible predictors and were used to develop the final linear random intercept models. The Šidák *p*-value correction was applied to selected features from cross-validation.

The Efron’s pseudo-R-square was calculated to measure how much variation of an output variable is explained by the independent variable(s). Mean Absolute Error (MAE) and Root Mean Squared Error (RMSE) measures were used to quantify the difference between predicted values and true values.

#### Approach B

The average features of all channels within the frontal, parietal, occipital, and temporal brain cortices were calculated. These specific brain regions have been extensively explored in research studies that aim to understand the intricate relationship between brain function and cognitive workload^102,103^. This process resulted in a set of 96 distinct features (i.e., six feature types across four brain cortices, each at four band frequencies). A widely used feature selection method, the Generalized Linear Mixed Model using penalized Lasso method (known as GLMM-LASSO), was used to develop models for performance and cognitive workload based on this feature set. The Local Outlier Factor (LOF) algorithm was used to detect outlier samples and exclude those from the analyses.

The Generalized Linear Mixed Model with a penalized Lasso method (known as GLMM-LASSO) was used to identify significant features. This penalized Lasso method simultaneously selects variables and estimates coefficients^104^. The tuning parameter, lambda, was meticulously chosen using the Bayesian Information Criterion (BIC) and cross-validation techniques.

#### Approach C—extension of methods to scenario where less density EEG system is used

Reducing the number of electrodes used in EEG collection is a growing trend in practical applications, such as evaluating or training the cognitive workload and performance of pilots/aviators in a simulator^105^. Thirty-two EEG channels, from the original 116, were selected based on the international 10–20 system. Configurations employing fewer than 32 channels may significantly compromise spatial resolution, potentially resulting in decontaminated EEG signals that do not fully reflect brain activity at their respective locations^106^.

Signals from 32 channels were analyzed, with features extracted using functional brain network and spectral analysis approaches. Average features were then extracted for channels within the frontal, parietal, occipital, and temporal brain cortices. This process resulted in a set of 96 distinct features. Importantly, irrespective of the specific configuration of an EEG headset, all inherently have some electrodes associated with these cortices. Outliers were identified and excluded from the analyses using the LOF algorithm. Finally, we employed the GLMM-LASSO to develop models for performance and cognitive workload based on this feature set.

### Reporting summary

Further information on research design is available in the Nature Research Reporting Summary linked to this article.

### Supplementary information

Supplementary table 1

Reporting Summary

## Data Availability

The data analyzed in the current study are available at: Shafiei, S. B., Shadpour, S., Mohler, J., Seilanian Toussi, M., Doherty, P., & Jing, Z. (2023). Electroencephalogram and eye-gaze datasets for robot-assisted surgery performance evaluation (version 1.0.0). PhysioNet.

## Code availability

No custom code or mathematical algorithm was developed for this study. Details regarding the specific codes used can be found in the references cited. Any access restrictions or licensing information associated with the codes used can be obtained from the respective sources as indicated in the references.
