# Supplementary material for: Developing cognitive workload and performance evaluation models using functional brain network analysis
Source: NPJ Aging. 2023 Oct 6;9(1):22. doi: 10.1038/s41514-023-00119-z (PMC10558559; doi:10.1038/s41514-023-00119-z)
Supplement: Supplementary file 1 — Supplementary table 1 [file 41514_2023_119_MOESM1_ESM.pdf]

Supplementary table 1 Results of linear random intercept analysis for performance and cognitive workload evaluation at Matchboard levels 2 and 3 based on approach A (84 PSD features extracted from 116 EEG signals).

Performance, Matchboard 2, PSD features, Approach A			
Predictors	Estimate	Standard error	p-value
Average PSD in BA 45 at beta-band frequencies	-0.82	0.22	< 0.001
Subject was a significant random effect (p-value= 0.002); pseudo R^2 = 0.74; MAE=6.44; RMSE=8.83			
Cognitive workload, Matchboard 2, PSD features, Approach A			
Predictors	Estimate	Standard error	p-value
Average PSD in BA 40 at alpha-band frequencies	-0.41	0.12	0.002
Subject was a significant random effect (p-value= 0.001); pseudo R^2 = 0.96; MAE=2.10; RMSE=2.96			
Performance, Matchboard 3, PSD features, Approach A			
Predictors	Estimate	Standard error	p-value
Average PSD in BA 20 at beta-band frequencies	-0.49	0.11	< 0.001
Subject was a significant random effect (p-value= 0.04); pseudo R^2 = 0.46; MAE=10.20; RMSE=12.94			
Cognitive workload, Matchboard 3, PSD features, Approach A			
Predictors	Estimate	Standard error	p-value
Average PSD in BA 45 at beta-band frequencies	1.75	0.41	< 0.001
Subject was a significant random effect (p-value= 0.002); pseudo R^2 = 0.85; MAE=4.46; RMSE=6.69			
